# Intermittent preventive treatment of malaria in pregnancy: a cross-sectional survey to assess uptake of the new sulfadoxine–pyrimethamine five dose policy in Ghana

**DOI:** 10.1186/s12936-017-1969-7

**Published:** 2017-08-10

**Authors:** Ivy Owusu-Boateng, Francis Anto

**Affiliations:** 1 School of Public Health, University of Ghana, Legon, Ghana; 2 Civil Service Polyclinic, Accra, Ghana

**Keywords:** Malaria, Pregnancy, Intermittent preventive treatment, Sulfadoxine–pyrimethamine

## Abstract

**Background:**

Malaria in pregnancy poses a great risk to both mother and fetus. In Ghana, malaria accounts for 3.4% of deaths and 16.8% of all hospital admissions in pregnant women. In 2014, Ghana updated her policy on intermittent preventive treatment of malaria in pregnancy with sulfadoxine–pyrimethamine (IPTp-SP) to reflect the updated policy of the WHO. This study determined the level of uptake of sulfadoxine pyrimethamine (SP) to serve as baseline for monitoring progress and also reviewed stock levels of SP, a key factor in the programme implementation.

**Methods:**

A cross-sectional hospital-based study was carried out among nursing mothers who had delivered within 12 weeks and were seeking postnatal care at Osu Government Maternity Home in Accra. Antenatal record books of the mothers were reviewed and data collected on number of visits and receipt of IPTp-SP. Mothers were interviewed and data collected on their background characteristics and obstetric history. Data on SP stock levels for the past 6 months were also reviewed. Logistic regression analysis was carried out to determine antenatal indicators on uptake of IPTp-SP using Stata version 12.

**Results:**

The proportion of uptake of three-five doses of SP were: IPT3 (87.5%), IPT4 (55.7%) and IPT5 (14.5%). The proportion of women who received the first dose of SP at 16 weeks of gestation was 21.3%. Women who made ≥4 visits were more likely to receive ≥3 doses of SP than those who made <4 visits (AOR = 4.57, 95% CI 1.15–18.16, p < 0.05). Women receiving the first dose of SP in the third trimester were less likely to receive ≥3 doses of SP than those who received the drug in the second trimester (AOR = 0.04, 95% CI 0.01–0.16, p < 0.05). Stock levels of SP were adequate to meet the demands by the pregnant women at the Maternity Home for the period under review.

**Conclusions:**

The uptake of ≥3 doses of SP was high in the study area. Frequent visits to the antenatal clinic and early uptake of the first dose of SP by pregnant women are necessary to achieve the new target of five or more doses of SP.

**Electronic supplementary material:**

The online version of this article (doi:10.1186/s12936-017-1969-7) contains supplementary material, which is available to authorized users.

## Background

Malaria is a life-threatening infectious disease caused by parasites of the genus *Plasmodium* and transmitted through the bites of infected female *Anopheles* mosquitoes. There are currently five known *Plasmodium* species (*Plasmodium falciparum*, *Plasmodium malariae, Plasmodium vivax. Plasmodium ovale* and *Plasmodium knowlesi*) that cause malaria in humans. Two of these species—*P. falciparum* and *P. vivax* cause the most disease the world over. *Plasmodium falciparum* is the most prevalent malaria parasite on the African continent and responsible for most malaria-related deaths globally [1].

Malaria infection during pregnancy is a major public health problem, with serious consequences not only to the pregnant woman, but her fetus and the neonate, if not prevented or treated early. Intermittent preventive treatment of malaria in pregnancy (IPTp) using sulfadoxine–pyrimethamine (SP) is one of the strategies for preventing malaria in pregnancy. It involves the administration of a full treatment dose of SP to pregnant women at routine antenatal care (ANC) visits, regardless of whether the recipient is infected with malaria parasites or not [2]. Uptake of IPTp-SP is known to reduce the number of maternal malaria episodes, maternal and fetal anaemia, placental parasitaemia, and improves birth weight [3], as well as reduce neonatal mortality.

In 2012, the World Health Organization (WHO) updated the recommendations for IPTp-SP and now requires that, SP should be given to all pregnant women at each ANC visit until delivery. SP administration should commence early in the second trimester, with doses given at least 1 month apart [4]. The number of ANC visits made is a major determinant of IPTp-SP uptake [5]. Although a high proportion of pregnant women currently go for ANC services in some communities [6], in others, many are still unable to make the recommended four or more visits [7]. Recent studies still show that the level of uptake of two or more doses of SP is still low in some countries even after the new WHO policy.

There are still issues of stock-out and non-adherence to protocols by health care providers [8]. Several other service related and community factors such as unavailability of skilled attendants at ANC [9], poor attitudes of staffs and travel distance to health facilities still hinder the implementation of IPTp-SP [10]. In some cases, the women are given the drug and yet they do not swallow it as it is not always given as directly observed therapy [8]. Reports of low IPTp-SP coverage in many endemic countries in Africa [5, 6, 11] raise concerns about how to achieve the higher targets set in the new WHO policy.

The Ghana National Malaria Control Programme [12] also updated her policy and now recommends a minimum of five doses of SP. The SP is to be given at monthly intervals starting from 16 weeks of gestation until delivery. The implementation of this policy started in 2014. The old policy which was implemented in 2003, recommended three doses of SP, starting from 16 weeks and given before 36 weeks of gestation. The Strategic Plan for Malaria Control in Ghana, 2005–2015 had the objective to reach 100% of pregnant women with SP by 2015. Thus all pregnant women should be put on SP to prevent malaria in pregnancy.

Achieving the target (three doses) set in the old policy was a problem as IPT3 coverage was generally low over the years throughout the country, from the northern to the southern parts of the country. Studies in the Tamale Metropolis, in the northern sector reported IPT3 coverage of 46% in 2011 [13]; reports from the Ashanti region in the middle sector also indicate a low coverage of IPT3 of 37% [14] and a much lower coverage for the southern sector of 26% [15].

The purpose of the current study was, therefore, to establish the level of uptake of IPTp-SP under the new policy at the Osu Government Maternity Home, in the capital of Ghana, Accra at the start of implementation of the new policy to serve as baseline for the eventual evaluation of the programme in the metropolis and also review stock levels of SP, a key factor in the programme implementation. Using data from different levels—primary and secondary could provide a comprehensive baseline on which future evaluation can be done.

## Methods

### Study area

The study was carried out at the Osu Government Maternity Home in the Osu Klottey sub-district of Accra Metropolitan Area of Ghana. It is located in the eastern part of the city of Accra and covers an area of approximately 6.59 km^2^. There are seven Government health facilities and 18 private ones in the sub-district. The Government facilities consist of one hospital, one polyclinic and five clinics. The Osu Maternity Home is one of the five Government clinics in the sub-district. The catchment area of the facility has a population of about 47,900. Women in their fertility age (15–49 years) form about 36% of the total population in the catchment area.

Services offered in the facility include family planning, antenatal care, delivery, postnatal care, child welfare clinic, laboratory and pharmacy services. The ANC service is offered daily except on Wednesdays. The delivery service covers a 24-h period. There are nine beds in the lying-in ward and four beds in the labour ward. The postnatal and child welfare clinics are held on Wednesdays and Tuesdays respectively. There are twenty-four staffs at the facility made up of one nursing officer, nine midwives, one pharmacy technician, one laboratory technician, four community health nurses and eight supporting staffs. The 2013 annual report indicates that there were a total of 3337 ANC attendants and 963 postnatal and child welfare clinic attendants.

### Study design

A descriptive cross-sectional study was conducted among nursing mothers who had delivered within 12 weeks before data collection and were visiting the child welfare (CWC) or postnatal clinics of the Osu Government Maternity Home for health Study participants were consecutively recruited on daily basis until a predetermined sample size was obtained. The first mother to report at the CWC and postnatal clinics and every other mother who reported at the two units for care on the days of data collection were approached for possible inclusion in the study. The data collection was carried out over a period of 6 weeks during the months May and June, 2015.

### Sample size estimation

The sample size for the study was calculated using Cochran’s formula for finite or small populations (n = n_o_ **/**1 + [(n_o_ − 1)/N]). n_o_ was determined using the formula: Z^2^pq**/**e^2^ (for large populations) [16]. Where, n_o_ = estimated sample size for large populations, Z = 1.96 at 95% confidence interval, p = 33.6% (IPT3 coverage for Osu Government Maternity Home in 2013) [17], e = precision level of 0.05 and q = 1 − p. An estimated n_o_ of 343 was arrived at (assuming the population was large). Finite population adjustment was done to reflect the small population of mothers who received IPT3 in 2013 at the facility, using n = n_o_ **/**1 + [(n_o_ − 1)/N]. Where, n = required sample size, n_o_ = estimated sample size for large population and N = the population size (963). The required sample size estimated was 253.

### Inclusion/exclusion criteria

All nursing mothers who had attended the Osu Government Maternity Home for ANC services during their most recent pregnancy and gave written informed consent to participate in the study were eligible to participate. Nursing mothers who had delivered beyond 12 weeks at the time of data collection were excluded to minimise the problem associated with recall.

### Data collection procedure

Data on socio-demographic characteristics such as age, educational level, number of children, occupation and marital status were collected directly from the nursing mothers onto a data collection form designed specifically for this study. Also, data on ANC services provided including whether SP was available for them at the ANC clinic, the number of tablets swallowed per dose and whether the drug was administered under supervision were collected directly from the mothers. For the purpose of accuracy, data on gestational age at first ANC visit, number of ANC visits during their last pregnancy, number of doses of SP taken before delivery and the gestational age at which first dose of SP was taken, were extracted from the ANC record books of the mothers.

Data on SP receipts, stock levels and stock outs for the past 6 months were collected from the pharmacy records of the health facility using a data extraction form. Antenatal registers at the clinic were also reviewed for daily issuing of SP to eligible pregnant women. The data collection involved face-to-face interview of the mothers. This was carried out by trained research assistants with university degrees, who are fluent in the local languages most used in area (Ga and Twi) and English. The questionnaire was in English and so the information was recorded in English. Data on SP stock levels were collected by a trained pharmacist (The list of variables measured is shown in Table 1).

### Quality control

Quality control was conducted by pre-testing the questionnaire to determine its appropriateness and suitability for the study. This resulted in corrections, rephrasing of questions and rearrangement of sections in the questionnaire. Pre-testing was done using 20 ANC attendants over a period of 2 days (10 per day) at the Civil Service Clinic also in the Osu-Klottey district and with similar health services. To ensure uniformity of the process, the two data collectors involved in the study were trained for 5 days on how to explain the study objectives and conduct the interviews and obtain informed consent. Data extracted from the ANC books were verified by a supervisor at the facility.

### Data processing and analysis

Data entry was done in Microsoft Excel software version 2013, cross-checked for completeness and imported into Stata version 12 for cleaning and analysis. The data were summarized using descriptive statistics including frequencies, percentages, means, standard deviation, median and ranges. The uptake of IPTp-SP was categorized into <3 doses versus ≥3 doses (based on the minimum required doses recommended in the earlier Ghana IPTp-SP policy). The socio-demographic and ANC characteristics were also grouped into categories. Chi square/Fischer Exact tests were conducted to establish association between uptake of IPTp-SP and each independent categorical variable. Any association with a p < 0.05 was considered significant. Logistic regression analysis reporting odds ratio was used to determine the strength of association between uptake of IPTp-SP and any significant independent variable that was found after the Chi square test.

Data collected from the pharmacy were also summarized using descriptive statistics. The number of tablets of SP per month that were less than the minimum stock level were categorized as not adequate and those that were more than the minimum stock level was categorized as adequate. Maximum stock level of the drugs was computed based on the rate of consumption of the drug by the clients at the facility taking into account the daily attendance at the facility.

**Table 1:** List of variables measured

Variables	Operational definitions	Type of variable
Outcome variable		
Uptake of IPTp-SP	Doses of SP received during pregnancy	Binary variable
Independent		
Socio-demographic, characteristics		
Age	The age in years of the woman	Continuous variable
Marital status	Married or not married	Categorical variable
Level of education	Stage of education attained	Categorical variable
Occupation	Self employed or government employed or unemployed	Categorical variable
Number of children	The number of live births of the woman	Continuous variable
Number of ANC visits	The number of visits to ANC during last pregnancy	Continuous variable
Gestational age at first dose of SP	Stage of pregnancy in weeks at receiving first dose of SP	Continuous variable
Gestational age at first ANC visit	Stage of pregnancy in weeks at first ANC visit	Continuous variable
Where did you get SP drug?	Place where SP was dispensed to pregnant woman	Categorical variable
Was the drug taken under a Nurse’s observation?	Taking SP under supervision of health worker	Binary variable
How many tablets did you swallow	Number of tablets of SP swallowed per dose	Categorical variable
Stopped folic acid whiles taking SP	Suspension of folic acid whiles taking SP	Binary variable
Stock level of SP	The quantity of SP tablets in stock	Continuous variable
Stock outs of SP	The number of days that drug was not available at the facility	Continuous variable
Source of SP	The place of procurement of SP drug	Categorical variable
Frequency of issue of SP	The number of times of issue of drug to ANC	Continuous variable
Challenges of stocking of SP	The problems associated with procurement of SP	Binary variable

## Results

### Characteristics of study participants

A total of 255 nursing mothers, aged 15–47 years (mean: 27.1 years; SD: 5.5) participated in the study. Most of the participants (65.1%; 255/166) were aged 20–29 years, with majority (83%) of them married. The highest level of education for 44.7% of them was basic education, with 29 (11.4%) having no formal education. Majority of the participants (208, 81.6%) were engaged in some form of employment. The mean number of children was 2.0 (range 1–7; SD: 1.1) (Table 2).

**Table 2:** Background characteristics of study participants

Characteristics	Frequency (n)	Percentage (%)
Age		
15–19	10	3.9
20–29	166	65.1
30–39	73	28.6
40–47	6	2.4
Marital status		
Married	211	82.8
Single	44	17.2
Educational level		
No formal education	29	11.4
Basic education	114	44.7
Secondary education	80	31.4
Tertiary education	32	12.5
Occupation		
Employed	208	81.6
Unemployed	47	18.4
Number of children		
1–2	175	68.6
3–4	72	28.2
5–7	8	3.2

### ANC attendance and IPTp-SP uptake

Most of the study participants (49.4%, 126/255) registered for the first ANC visit in the second trimester of their pregnancy, with only 9.4% (24/255) registering during the third trimester. The mean gestational age at first ANC visit was 15.9 weeks (SD: 6.4; range 4–34 weeks). The number of ANC visits made ranged from 1 to 9, with a mean of 4.9 (SD: 1.4). A total of 226 (88.6%) of the mothers made four or more visits, 10 (3.9%) made eight or more visits, whilst 2 (0.8%) made nine visits before delivery (Table 3).

**Table 3:** ANC attendance and IPTp-SP uptake among study participants

Characteristics	Frequency (n = 255)	Percentage (%)
Gestational age at first ANC		
First trimester	105	41.18
Second trimester	126	49.41
Third trimester	24	9.41
Number of ANC visits		
<4	29	11.37
≥4	226	88.63
Number of doses received		
None	3	1.18
One dose	10	3.92
Two doses	19	7.45
Three doses	81	31.76
Four doses	105	41.18
Five doses	37	14.51
Gestational age at first dose of SP		
16	53	21.03
17–24	165	65.48
25–36	34	13.49
Number of SP tablets swallowed per dose during pregnancy		
2	3	1.2
3	249	98.8
Place where SP was dispensed		
ANC	250	99.21
Pharmacy	2	0.79
Stopped folic acid whiles taking SP drug		
Did not stop	249	98.8
Stopped	3	1.2
Took SP drug under DOT		
Not directly observed	2	0.8
Directly observed	250	99.2

*n* number of respondents, *ANC* antenatal centre, *SP* sulphadoxine pyrimethamine, *DOT* direct observed therapy

Only 3 (1.2%) of the mothers did not take SP during their most recent pregnancy giving IPTp coverage of at least one dose of 98.8% (252/255). Of the three who did not take the drug, two made their first ANC visit during the first trimester and made a total of six visits each, while the third mother made the first visit during the third trimester and made a total of two visits to the ANC clinic before delivery. Two hundred and forty (94.1%) respondents had babies weighing 2.5 kg or more with mean birth weight of 3.1 kg (SD: 0.4) and ranging from 2.1 to 4.8 kg (Table 4).

**Table 4:** Relationship between ANC characteristics, socio-demographic characteristics and IPTp-SP uptake among recently delivered women

Variables	Frequency	IPTp-SP uptake (%)		p value
	(n = 255)	<3 doses	≥3 doses	
Gestational age at first ANC				
First trimester	105	6.67	93.33	
Second trimester	126	9.52	90.48	<0.001
Third trimester	24	54.17	45.83	
Number of ANC visits				
<4	29	44.28	51.72	<0.001
≥4	226	7.96	92.04	
Gestational age at first dose of SP				
16	53	0.00	100	
17–24	165	6.67	93.33	<0.001*
25–36	34	52.94	47.06	
Number of children				
1–2	175	10.29	89.71	
3–4	72	16.67	83.33	0.16*
5–7	8	25.00	82.5	
Marital status				
Married	211	11.37	88.63	0.22
Single	44	18.18	81.82	
Educational level				
No formal education	29	10.34	89.66	
Basic education	114	17.54	82.46	0.22*
Secondary education	80	8.75	91.25	
Tertiary	32	6.25	93.75	
Occupation				
Employed	208	11.54	88.46	0.31
Unemployed	47	17.02	82.98	
Age group				
15–19	10	10	90	
20–29	166	15.06	84.94	0.44*
30–39	73	8.22	91.78	
40–47	6	0.00	100	
Weight of child at birth (kg)				
<2.5	15	60	40	<0.001
≥2.5	240	9.58	90.42	

*IPTp*-*SP* intermittent preventive treatment in pregnancy with sulphadoxine pyrimethamine, *n* number of respondents

* Fischer’s exact value

**Fig. 1:**
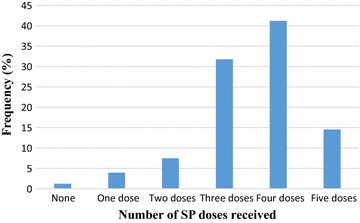
IPTp-SP uptake among recently delivered women at Osu Government Maternity Home, Accra

Most of the mothers received three or four doses (73.8%) of SP with only 4.0% (10/252) of them receiving a single dose (Fig. 1). This gives the proportions of IPTp coverage of IPT1, 98.8%; IPT2, 94.9%; IPT3, 87.5%; IPT4, 55.7% and IPT5, 14.5%. Categorizing the extent of IPTp-SP coverage into two groups, 88.5% (223/252) received three or more doses whiles 56.3% (142/252) received four or more doses (Additional file 1).

Pearson’s Chi square/Fischer’s exact test revealed that, gestational age at first ANC visit, total number of visits to the ANC and gestational age at receiving the first dose of SP were significantly associated with uptake of IPTp-SP (p < 0.001). None of the socio-demographic characteristics was found to be associated with IPTp-SP uptake (p > 0.05) (Table 3).

Univariate logistic regression analysis (Table 4), revealed that IPTp-SP uptake of ≥3 doses during pregnancy was six times less among respondents registering their first ANC in the third trimester than those in first trimester (COR = 0.06, 95% CI 0.02–0.18, p < 0.001). Uptake of ≥3 doses was 10.76 times higher among women visiting the ANC for ≥4 times during pregnancy than those visiting the ANC for <4 times (COR = 10.7, 95% CI 4.5–25.82 p < 0.001). The odds of receiving ≥3 doses of SP was five times less in women receiving the first dose in the third trimester than those taking it in the second trimester (COR = 0.05, 95% CI 0.02–0.12, p < 0.001). After adjusting for other characteristics of the respondents, having ≥four visits (AOR = 4.57, 95% CI 1.15–18.16, p < 0.05) and gestational age at first dose of SP (AOR = 0.04 95% CI 0.01–0.16, p < 0.001) were significantly associated with receiving ≥3 doses of SP during pregnancy (Table 5).

**Table 5:** Effect of ANC characteristics of respondent on IPTp-SP uptake of ≥3 doses

Characteristics	IPTp-SP uptake		COR (95% CI)	p value	AOR (95% CI)	p value
	<3 doses n (%)	≥3 doses n (%)				
Gestational age at first ANC visit						
First trimester	7 (6.67)	98 (93.33)	Ref		Ref	
Second trimester	12 (9.52)	114 (90.48)	0.68 (0.26–1.79)	0.434	0.74 (0.22–2.46)	0.630
Third trimester	13 (54.17)	11 (45.83)	0.06 (0.02–0.18)	<*0.001*	2.8 (0.31–24.55)	0.352
Number of visits						
<4	14 (48.28)	15 (51.72)	Ref		Ref	
≥4	18 (7.96)	208 (92.04)	10.76 (4.5–25.82)	*<0.001*	4.57 (1.15–18.16)	*0.031*
Gestational age at first dose of SP						
Second trimester	11 (5.05)	207 (94.95)	Ref		Ref	
Third trimester	18 (52.94	16 (47.06)	0.05 (0.02–0.12)	*<0.001*	0.04 (0.01–0.16)	*<0.001*

*COR* crude odds ratio, *AOR* adjusted odds ratio, *95% CI* 95% confidence interval, *Ref* reference

Significant p values are presented in italics

### Gestational age at first dose of IPTp-SP

**Fig. 2:**
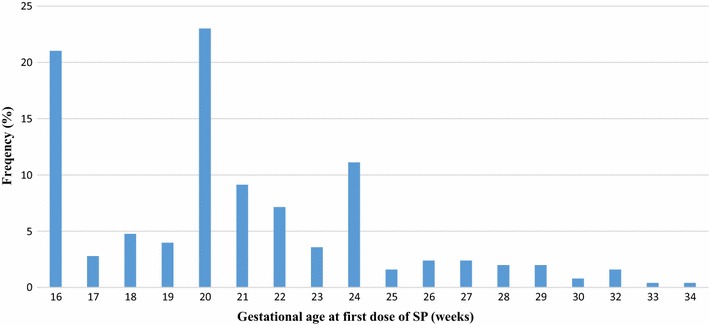
Gestational age at uptake of first dose of IPTp-SP among recently delivered women in Accra, Ghana

The median gestational age at which respondents received the first dose of SP was 20 weeks (SD: 3.9) ranging from 16 to 34 weeks. Of the total of 252 respondents who received SP, 53 (21.0%) received the first dose at 16 weeks, 4 (1.6%) at 32 weeks, with the majority of them (58, 23.0%) receiving the first dose at 20 weeks (Fig. 2). Gestational age at first dose of SP was associated with uptake of ≥3 doses of SP (p < 0.001). Most of the mothers (94.95%, 207/218) who took their first dose in the second trimester, received more doses. For those who received the first dose of SP in the third trimester, 47.1% (16/34) received ≥3 doses, whiles 18 (52.9%) received <3 doses of SP.

### Stock-levels of SP at the Maternity Home

**Fig. 3:**
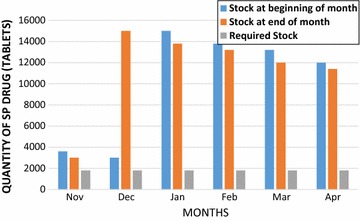
Stock levels of SP at the maternity home for 6 months period (Nov 2014–April 2015)

Stock-levels of SP at the pharmacy was found to be adequate and there was no stock-out of the drug throughout the period of review (Fig. 3). The drug was procured from the Regional Medical Store. About ninety-nine percent of respondents had their SP given to them by the midwives at the ANC clinic. This is an indication that SP was available at the clinic. A review of the ANC register, presented a daily dispensing of SP to all pregnant mothers who were eligible to receive the drug.

## Discussion

Results of the current study revealed that most of the study participants (90.6%) registered for their first ANC visit during the first or second trimester of pregnancy. Majority of them (88.6%) made at least four visits before delivery, as recommended by the WHO in the previous policy on ANC visits but only 3.9% made the required eight visits per the new policy [18]. Most of these women (56%) received four or more doses of SP with 86.5% of the first doses being taken during the second trimester. Stock-out of SP was not observed during the period under review (Fig. 3).

Antenatal care services are essential services designed to improve maternal and new born health. Although timely ANC visit is necessary for early detection and management of pregnancy related problems, many mothers do not receive such care [19] especially in low income countries and this could have negative consequences on overall perinatal outcomes. According to [20], trends in most sub-Saharan countries seem to suggest that most women do not have their first ANC visit during the first trimester. In the current study however, 41.2% of the mothers had their first ANC visit in the first trimester which was about twice that reported (20.5%) from Cameroon [20] and a much higher level reported (82.4%) from the Democratic Republic of Congo [21]. Thus, some improvement is being seen in Africa at the start of implementation of the new WHO policy.

Several individual, social and health service challenges have been identified as contributing to delay in initiating ANC visits. These challenges include, cost of service, distance to health facilities and waiting time for services [21]. Others include, lack of correct knowledge of the recommended ANC schedule [22], poverty and low level of education [20].

Appropriate interventions such as free ANC services, efficient mutual health insurance schemes, improved road network and more user-friendly educational programmes, that will target both men and women of childbearing age, may help address these challenges [21]. When mothers receive early ANC services and report for all scheduled clinics, the new target of eight ANC visits can be achieved even in low-income countries, leading to improved maternal and new born health. This is because IPTp-SP can reduce maternal malaria [23] as well as episodes of clinical malaria in the early years of life [24].

Though very important, the gestational age at which a pregnant woman made the first ANC visit was not the main determinant of receiving more doses of SP in the current study, but rather the number of visits made before delivery as reported in some earlier studies [5, 25, 26]. The current policy of giving SP early in the second trimester till delivery is, therefore, laudable as even those who start late but continue till delivery will avail themselves for more doses of SP [5]. Thus, allowing for continuous uptake of SP till delivery instead of stopping before 36 weeks gestation (as the case was in the previous policy of Ghana), allows for late registrants to avail themselves for more doses. Earlier studies in Kenya by Hill and colleagues [27] however, reported higher doses of SP being received by women who reported in the first trimester. This happens if the women report for all scheduled ANC clinics, and that should be encouraged.

Another key determinant of receiving more doses of SP as revealed in the current study was the time the first dose was received [28]. Receiving the first dose early in the second trimester allowed for more doses to be taken. This brings to focus the schedule of ANC visits, which requires mothers to report on monthly basis. Thus, for instance, if a mother comes for ANC during week 15, she would be required to report for the next ANC during week 19, thereby delaying the time of uptake of the first dose of SP. Such mothers could be scheduled to report after one or 2 weeks instead of four, to receive the first dose. In most cases, this is possible when adequate education is given to the mother.

Stock levels of SP at the Osu Government Maternity Home was found to be adequate as the quantity of SP at the beginning and end of each of the 6 months review period were above the required stock levels for the facility, based on the number of clients. Shortage of SP at facilities have been identified as a barrier to achieving high IPTp-SP coverage [13, 29–32] in many earlier studies. Though two mothers missed the opportunity to receive SP in the current study, it was not likely due to stock-out of the drug. The main issue identified was the fact that SP stocked levels were above the levels required for the facility which could have financial implications if the drug should get expired.

## Conclusions

The current study has revealed a significant improvement in uptake of SP during the second year of implementation of the programme. Frequent visits to the ANC clinic and early uptake of the first dose of SP were necessary to achieve high coverage. Pregnant women should, therefore, be encouraged to register early for ANC services and report for all scheduled clinics. Follow-up studies including qualitative studies involving stakeholders are recommended at 2–5 years interval to monitor progress of implementation of the guidelines and outcomes in terms of episodes malaria and birth outcomes and not wait till 2030.

## Limitation of the study

The study collected information on SP administration and ANC services offered to mothers during their last pregnancy. There is a possibility of recall bias, however, recall was limited to only 3 months which might not affect the reliability of responses given. Also, the ANC books were used to validate most of the information required. A larger sample size might also be helpful in explaining further some of the observations made, however, the sample size was powered enough to represent the study population and serve as a baseline for future monitoring.

## Additional file

**Additional file 1.** IPTp uptake at Osu Government Maternity Home, Accra.

## Abbreviations

ANC: antenatal care
CWC: Child Welfare Clinic
IPT: intermittent preventive treatment
IPTp-SP: intermittent preventive treatment of malaria in pregnancy with sulfadoxine–pyrimethamine
NMCP: National Malaria Control Programme
PMI: President’s Malaria Initiative
SP: sulfadoxine–pyrimethamine
